# A Comprehensive Study on Peripartum Hysterectomy in Tehran, Iran: Causes and Clinical Outcomes

**DOI:** 10.34172/aim.33449

**Published:** 2025-06-01

**Authors:** Fahimeh Ghotbizadeh Vahdani, Sedigheh Hantoushzadeh, Zahra Panahi, Mohammad Haddadi, Mamak Shariat, Zahra Tavoli, Fatemeh Asadi, Arezoo Maleki, Zeinab Mansouri, Marjan Ghaemi

**Affiliations:** ^1^Maternal, Fetal & Neonatal Research Center, Family Health Research Institute, Tehran University of Medical Sciences, Tehran, Iran; ^2^Vali-E-Asr Reproductive Health Research Center, Family Health Research Institute, Tehran University of Medical Sciences, Tehran, Iran

**Keywords:** Peripartum hysterectomy, Placenta accreta spectrum, Public health, Uterine atony

## Abstract

**Background:**

Peripartum hysterectomy (PH) poses a notable public health challenge due to its correlation with severe maternal morbidity and mortality. Identifying the causes and risk factors associated with PH is crucial for enhancing preventive strategies and maternal health outcomes. This study investigates the indications, and outcomes of PH across healthcare settings.

**Methods:**

This is a retrospective analysis of all PH cases between March 2021 and March 2022 in Tehran, Iran. The study included cases where gestational age was greater than 24 weeks, with PH performed during delivery or within the first 24 hours postpartum. Data, including demographic and clinical variables, were collected from the Iranian Maternal and Neonatal Network (IMaN) system.

**Results:**

In total, 136 PH cases were identified. The average age of the women was 35.1 (±5.2) years. Cesarean section (C/S) was the predominant delivery method, comprising 93.4% of cases, with 44.9% having a medical indication, 7.4% without indications, and 41.2% performed as emergency C/S. Notably, a previous C/S was reported in 65.4% of cases. The main indications for PH were placenta abnormalities (75.6%), uterine atony (20.7%), uterine rupture (3%), and ovarian mass (0.7%). Placenta previa (42.6%) and placenta accreta (32.4%) were the most frequent placental abnormalities.

**Conclusion:**

The findings highlight key factors influencing PH that may inform health policies to mitigate risks. A national study could offer a comprehensive view, accounting for regional, socioeconomic, and cultural differences, emergency healthcare access, and resource availability, guiding targeted public health interventions.

## Introduction

 Peripartum hysterectomy (PH) is a major surgical procedure and includes hysterectomies performed either during or within 24 hours after delivery.^1^ PH is typically performed in cases of unmanageable massive obstetric hemorrhage following abnormal placentation, uterine atony, uterine rupture, or puerperal sepsis.^1^ The incidence of PH worldwide is approximately 1.1 per 1000 deliveries, varying by country, with higher rates in lower middle–income countries than high-income countries.^1^

 PH can be performed electively, such as when abnormal placentation is diagnosed before delivery or for cervical cancer detected during pregnancy that is a candidate for surgery. It is, however, more commonly performed as an emergency procedure following vaginal or cesarean delivery.^2^ Risk factors include advanced maternal age, placental abnormalities, high parity, and a history of cesarean sections.^3^

 The World Health Organization reports that more than 1 in 5 births, or 21%, worldwide undergo cesarean sections. It is estimated that this percentage could increase to even 29% over the following decade.^4^ As the rate of cesarean sections increases, there is a higher proportion of placental abnormalities, such as placenta previa and placenta accreta, which further explain the increased frequency of PH.^5,6^

 PH is associated with high levels of maternal morbidity and mortality, as well as many short- and long-term complications. Short-term complications include additional surgical procedures, bladder or ureter damage, massive bleeding requiring blood transfusion, fever, and infection.^1,2^ Although loss of fertility is indicated in most studies as one of the long-term complications, a cohort study showed that women who had undergone PH had more problems during the first year after delivery compared to those without a PH history. These challenges included difficulties in bonding with their newborn, pain, depression, problems or pain during intercourse, fatigue or extreme tiredness, flashbacks to labor or birth, menopausal symptoms, and difficulties with concentration.^7^

 PH can have considerable economic impacts, creating a substantial financial burden for hospitals and healthcare providers. A study of PH-related costs found the procedure to be associated with increased health care costs in the first five years post-delivery. This increase was more pronounced in raised costs of hospitalizations in the first year. This translates to £5380 in additional primary and secondary care in the first five years post-delivery compared with women who had not undergone a hysterectomy. The research indicated that, on average, women who had PH required more consultation sessions and had twice as many prescriptions and medical tests at primary care facilities in the first year postpartum compared to those who did not undergo the procedure. Furthermore, a higher proportion of women in the hysterectomy group had more outpatient visits, hospital admissions for non-delivery-related conditions, and longer average hospitalization.^8^

 As PH poses a challenge to mothers, has a significant economic burden on the healthcare system, and given the high rate of cesarean sections in Iran that amplifies the risk of PH,^9^ comprehensive strategies and policies to prevent and control PH should be formulated. So, in this regard, one of the fundamental prerequisites is an understanding and recognition of the underlying determinants of PH. As a result, this helps the clinician better explain the risks to which the patient is possibly exposed. Besides, policymakers can also enforce strategic planning and policy guidelines at health systems to reduce the prevalence of unnecessary cesarean sections throughout the nation. Therefore, this study aims to analyze PH in Tehran, as a metropolitan city with many health centers in Iran, focusing on its causes, indications, and outcomes.

## Materials and Methods

###  Study Design and Population

 This is a retrospective cross-sectional study involving all cases of PH in Tehran, Iran, from March 2021 to March 2022. The inclusion criteria were PH at any gestational age, PH performed during delivery or within the first week postpartum, and delivery conducted in private and public hospitals in Tehran. Incomplete medical records were considered an exclusion criterion.

 The study complied with the principles of the Declaration of Helsinki,^10^ taking into account patient confidentiality and anonymity. Demographic and clinical data were collected from the medical records. The Ethics Committee of Tehran University of Medical Sciences approved the study, and the project is registered under the ethics code IR.TUMS.IKHC.REC.1401.219 in the National Ethics System. Informed consent was not obtained from individuals because it was a retrospective study based on documented information, and so it was waived by the Ethics Committee.

###  Data Collection 

 We obtained demographic and clinical information from the medical records of individuals registered in the Iranian Maternal and Neonatal Network (IMaN) system. This information was registered on a checklist designed by the researcher and implemented in Microsoft Excel. The IMaN is an overarching network for the surveillance of maternal and neonatal health. It electronically registers almost all the information concerning childbirth, such as demographic data and health information of mothers and neonates, from all of the country’s hospitals. Since 2014, IMaN has recorded not only hospital births, but also all births occurring outside of healthcare facilities, including those at home, in maternity centers, or other locations.^11^

 The demographic variables measured in this study were mother’s age, body mass index (BMI), parity, gravidity, education, gestational age, birth weight, conception method, singleton/multiple pregnancies, prenatal care visits, uterine surgery history, mother’s comorbidity, and type of hospital. Clinical data encompassed type of delivery, indications for surgery, placental abnormality including placenta previa and placenta accreta spectrum (diagnosed by ultrasonography or MRI),^12^ time interval from complication to PH, operation time, admission time and discharge time hemoglobin (Hb) levels, white blood cell count (WBC), platelet (PLT) levels, need for post-operative blood transfusions, maternal mortality and morbidity, and receiving iron supplements.

 We assessed the relationship between certain demographic and clinical variables and placental abnormalities by comparing cases with and without abnormal placentation, based on prenatal ultrasound or MRI findings.

###  Statistical Analysis 

 SPSS version 25 was used to analyze the data for this study. Quantitative data were shown as mean and standard deviation, while qualitative data were in percentages and numbers. The chi-square test was used to determine the association of placental anomalies with selected demographic and clinical variables. Binary logistic regression analysis examined the relationship between some confounder variables with placental anomaly with the significance level set at 0.05.

## Results

 Among 173 cases of PH, 136 (78.61%) women with complete medical records were included in the study. Demographic characteristics are summarized in Table 1. The mean age was 35.10 ( ± 5.2) years, and the mean BMI was 29.74 ( ± 4.76). The mean gravidity and parity were 3.46 ( ± 1.56) and 1.84 ( ± 1.12), respectively. The most common conception method was spontaneous pregnancy (91.9%), 7.4% of women became pregnant using ART, and 94.7% of them were singleton pregnancies. Public hospital clinics (29.4%) and private offices of specialists (19.9%) provided more prenatal care to mothers, and most childbirths were done in public hospitals by residents of obstetrics and gynecology. A previous caesarian section was done in 65.4% of females. In 23 women, comorbidity was positive, with hypothyroidism (n = 8), diabetes mellitus (n = 4), hyperthyroidism (n = 3), heart disease (n = 2), and hypertension (n = 2) being the most frequent diseases. The mean birth weight and gestational age were 2645.96 ( ± 695) grams and 34.94 weeks.

**Table 1:** Demographic Characteristics of the Studied Women

**Variables**		**Number**	**Valid Percent**
Education	Illiterate	14	10.3
	Primary school	17	12.5
	High school	20	14.7
	University	15	11.0
	Unknown	70	51.5
Conception method	Spontaneous	125	91.9
	ART	10	7.4
	Ovulation induction	1	0.7
Pregnancy type	Singleton	124	94.7
	Multiple	7	5.3
Uterus surgery history	No	36	26.5
	Myomectomy	2	1.5
	Curettage	5	3.7
	Cesarean section	89	65.4
	Others	4	2.9
Comorbidities	No	113	83.1
	Yes	23	16.9
Prenatal care	Without prenatal care	1	0.7
	Health center	24	17.6
	Public hospital clinic	40	29.4
	Private office of specialist	27	19.9
	Unknown	44	32.4
Hospital of delivery/Delivery attendant	Public hospital/specialist	40	29.4
	Public hospital/resident	69	50.7
	Public hospital/midwife	2	1.5
	Private hospital/specialist	22	16.2
	Private hospital/midwife	0	0
	Unknown	3	2.2
	**Min-Max**	**Mean**	**±Standard Deviation**
Age (y)	21-54	35.10	5.20
BMI	13.32-44-14	29.74	4.76
Gravidity	1-11	3.46	1.56
Parity	0-5	1.84	1.12
Gestational age (wk)	14-42	34.94	4.66
Birth weight (g)	700-4100	2645.96	695.0

ART, Assisted reproductive technologies; BMI, body mass index.

 As shown in Table 2, 93.4% of births occurred through a caesarean section, of which 44.9% had medical indications, 7.4% were done without indications, and 41.2% were performed as an emergency cesarean section. Indications of PH were placental abnormality (75.6%), uterine atony (20.7%), uterine rupture (3%), and ovarian cancer (0.7%). Among placentation anomalies, placenta previa (42.6%) and accreta (32.4%) were more frequent. The ICU/CCU admitted 51.5% of cases, with 15.4%, 7.4%, 7.4%, and 2.9% of mothers experiencing urinary tract injury, oophorectomy, re-surgery, and end-organ damage, respectively. In three cases, maternal death occurred. During pregnancy, 67.6% of the studied women took iron supplements. Blood transfusion was needed in 88.2% of the population. The time interval from complication to PH in 42.6% of mothers was during delivery, 30.9% in the first 6 hours postpartum, and 2.2% in the 6–24 hours postpartum. The mean number of admission days in intensive care units was 1.98. The mean serum Hb level at admission and discharge were 11.64 and 9.65 g/dL, respectively. The mean serum platelet and WBC levels at admission time were 201.61 and 13.47 ( × 10^3^/µL), respectively, while their discharge serum levels were 171.27 and 13.53 ( × 10^3^/µL), respectively.

**Table 2:** Clinical Characteristics of the Studied Women

**Variables**		**Number**	**Valid Percent**
Delivery type	Vaginal	9	6.6
	Elective C/S (previous C/S, malpresentation)	61	44.9
	Elective C/S (maternal request)	10	7.4
	Emergency C/S	56	41.2
Hysterectomy indication	Placental abnormality	102	75.6
	Atony	28	20.7
	Uterine rupture	4	3.0
	Ovarian cancer	1	0.7
Placental abnormality^*^	Accreta	44	32.4
	Increta	21	15.4
	Percreta	8	5.9
	Previa	58	42.6
Iron supplement	No	1	0.7
	Yes	92	67.6
	Unknown	43	31.6
Blood transfusions	No	16	11.8
	Yes	120	88.2
Hysterectomy time	During surgery	91	66.9
	First 6h	42	30.9
	First 6-24h	3	2.2
Maternal morbidity^*^	Urinary tract injury	21	15.4
	End organ damage	4	2.9
	Oophorectomy	10	7.4
	Re-surgery	10	7.4
	Iliac injury	1	0.7
	Admission in ICU/CCU	70	51.5
	**Min-Max **	**Mean**	**±Standard Deviation**
Admission time Hb (g/dL)	5.2-17.7	11.64	1.63
Discharge time Hb (g/dL)	4.1-14.7	9.55	1.50
Admission time WBC (10^3^/µL)	4.50-70.20	13.47	12.14
Discharge time WBC (10^3^/µL)	5.76-32.89	13.53	5.30
Admission time PLT (10^3^/µL)	35-436	201.61	66.78
Discharge time PLT (10^3^/µL)	56-364	171.27	65.23
Admission duration in ICU/CCU (day)	1-8	1.98	1.35

^*^Some patients had more than one placentation abnormality and maternal morbidity, which should be considered when accounting for these variables’ frequency.

 There was a significant association between abnormal placentation and maternal age (*P* = 0.005), gestational age (*P* < 0.001), multigravidity (*P* = 0.002), history of previous uterine surgery (*P* < 0.001), and hysterectomy time (*P* = 0.003). Patients with placental anomalies had higher material morbidity than those without an anomaly (*P* = 0.001) (Table 3).

**Table 3:** Association Between Placental Anomalies and Selected Demographic and Clinical Variables

**Variable**	**Abnormal Placentation**	**Normal Placentation**	***P*** ** Value**
Age (y)			
< 35	21	33	0.005
≥ 35	52	30	
Gestational age (wk)			
< 37	64	18	< 0.001
≥ 37	9	45	
Gravidity			
Primigravida	1	10	0.002
Multigravida	72	53	
Uterine surgery history			
No	4	32	< 0.001
Yes	69	31	
Blood transfusion			
No	11	5	0.198
Yes	62	58	
Hysterectomy time			
During surgery	57	34	0.003
Postpartum	16	29	
Maternal morbidity			
No	37	14	0.001
Yes	36	49	

 The regression analysis summary is shown in Table 4. An increase in the gestational age decreased the risk of placental anomalies by 15.5% (*P* = 0.03). Previous uterine surgery raised the risk of placental anomaly by a factor of 6.6 according to research data.

**Table 4:** Multivariable Logistic Regression Analysis of Factors Associated with Peripartum Hysterectomy

**Variable**	**Beta (β)**	**Adjusted OR**	**95% CI for OR**	***P*** ** Value**
Maternal age	0.087	1.091	0.998-1.192	0.055
Gestational age	-0.155	0.856	0.744-0.985	0.030
Gravidity	0.005	1.005	0.767-1.317	0.970
Uterine surgery history	1.896	6.662	1.926-23.044	0.003

## Discussion

 PH is considered one of the near-miss events critical for life-saving purposes in cases of peripartum massive hemorrhage. Key risk factors for PH include advanced maternal age, previous or current caesarian delivery, and abnormal placentations. The incidence of PH varies in different regions due to different factors, including the rate of caesarian sections, the protocols for managing obstetric emergencies, the availability of medical facilities and technology, and even more generally, the overall birth rate.

 In our study, the mean maternal age was 35.1 years ( ± 5.2), which is higher than previous reports, such as a mean age of 31.8 years from two central hospitals ^1^ and 32.4 years from another hospital^13,14^ in the different regions of the country. In a population-based study, maternal age ≥ 35 years was also found to be a significant hazard for PH.^3^

 Advanced maternal age has direct and indirect effects on the PH rate. With advancing age, fertility decreases, heightening the chances of conception through assisted reproductive technology (ART), which has been associated with an elevated risk of PH in the literature.^15^ Besides, the birth rate has shown a decrease with increasing maternal age, possibly causing the incidence of PH per live birth to appear relatively higher. Actually, other adverse outcomes of pregnancy, including cesarean section delivery, have been also shown to be more common in women of older maternal age, thus contributing to the increasing frequency of PH.^16^ This study’s inclusion of all hysterectomies performed in both tertiary and private hospitals in the capital allows for a more comprehensive understanding of the women’s mean age undergoing this procedure, providing a generalizable index. However, it is important to note that the mean age may vary across different cities.

 In our population study, the cesarean delivery rate was 93.4%. Among them, 44.9% had an indication for a cesarean section, such as a repeat cesarean section, multiple pregnancies, or malpresentation. Of these, 41.2% of the cesarean sections were classified as emergencies, and 7.4% were done without a clear medical indication following a maternal request. Only 6.6% of the births were by vaginal delivery. An epidemiological study on PH data from nine countries found that cesarian section was the mode of delivery in 77.1% of cases, with 58.7% of these planned.^17^ An Italian study on 500 women who underwent PH reported a cesarean delivery rate of 83.9% over two years.^3^ Another study in Iran revealed that among 33 cases of PH, the rate of cesarean delivery was 90.9%.^14^ These findings point to a serious concern, as the rate of cesarean sections recommended by the World Health Organization is 10%-15%, meaning most of these operations are unnecessary.^18^

 The trend of cesarean delivery is increasing in many countries, including Iran, and has turned into a public health issue. The rate of cesarean delivery is within the range recommended by the World Health Organization in only 14 countries.^18^ In Iran, the rate of cesarean sections has been on the increase from 40.7% in 2005 to 48% in 2014, with some private hospitals recording up to an 87% rate.^19^ Although the implementation of the health transformation plan has led to a reduction in cesarean section rates, the statistics remain quite high.^20^

 Moreover, women who underwent cesarean deliveries before the health transformation plan are still at risk of repeat cesarean sections in subsequent pregnancies. To address this issue, it is crucial to enhance the training and skills of obstetricians to support vaginal birth after cesarean and to improve access to facilities and technologies, such as embolization, that could reduce the need for hysterectomies.

 The findings of this study revealed that placental abnormalities were an indication for PH in 75.6% of cases, and uterine atony was responsible for 20.7% of cases. In accordance with our findings, a study conducted in a different province of Iran showed that placenta accreta and uterine atony were the most common indications for PH.^21^ A similar trend was observed in a multicentric study, with 34.8% of PH cases being associated with placenta-accreta spectrum disorders and 35.3% with uterine atony.^17^ In 318 PH cases, Knight et al reported a rate of more than 50% uterine atony and 38% abnormal placentation.^22^

 However, our study demonstrated a significantly higher prevalence of placental abnormalities. This shift in indications, with placental anomalies now being the major cause of PH rather than uterine atony, aligns with contemporary reports. The literature emphasizes the improvement of uterine atony management and treatment, as well as the growth of abnormal placentation in the context of the increasing number of prior uterine scarring.

 We found a significant association between the time to hysterectomy and abnormal placental invasion. Specifically, in cases with abnormal placental invasion, most hysterectomies were performed during delivery, or, in other words, as emergency procedures. In cases of abnormal placental invasion, due to the extensive bleeding involved, there is limited time for conservative management, leading to a shorter interval between the diagnosis of hemorrhage and hysterectomy. One of the most critical factors contributing to this is the increased number of previous cesarean deliveries and prior uterine scarring. Supporting this, our study revealed that increased gravidity was significantly associated with a higher frequency of placental anomalies, while a similar study found no association between gravidity and abnormal placental invasion (*P* = 0.7).^23^

 This discrepancy may be attributed to Iranian mothers’ higher prevalence of prior uterine scarring from previous cesarean deliveries. Therefore, patient counseling and raising awareness regarding the mode of delivery, particularly for women who plan for multiple pregnancies, are very important. This should be part of antenatal care.

 Our study showed that 62.5% of women experienced various morbidities, such as urinary tract injuries, organ damage, oophorectomy, reoperation, and intensive care unit admission. A systematic review of approximately 15 000 PH cases revealed that postoperative morbidities occurred in one-fourth of the mothers.^1^ While PH increases maternal morbidity, it has a smaller impact on maternal mortality. Three cases of death were observed in our study, which is consistent with a similar study that reported one maternal death out of 62 cases of PH.^23^ A multi-country study spanning 21 countries reported a lower incidence of maternal mortality in Asia compared to Africa (2% vs. 3%) because of a higher incidence of PH in Asian women (7% vs. 5%).^24,25^

 Given that PH significantly increases the demand for blood transfusions and blood products, occupancy of intensive care unit beds, need for healthcare personnel and specialized equipment, as well as maternal physical and emotional morbidity, it imposes a substantial economic burden on the healthcare system of any country.^26,27^ As a result, PH prevention and reduction are both imperative and provide a valuable opportunity for the assessment and refinement of regional health policies.

 Strategies aimed at reducing the rate of unnecessary cesarean sections, particularly primary cesareans, enhancing preoperative assessments, advancing obstetricians’ expertise in emergency management, promoting multidisciplinary approaches in managing obstetric emergencies such as postpartum hemorrhage, and improving the availability of critical facilities and equipment can play a crucial role in decreasing the incidence of PH and its associated morbidity. Additionally, it may be beneficial to explore alternative approaches to traditional hysterectomy methods, in order to reduce negative side effects and financial strain. We also discovered that older mothers, with greater prior uterine scarring, and more caesarean deliveries in the study population are all factors that make PH more likely.

 This study investigates the characteristics of PH conducted in multiple centers in Tehran; however, several limitations have been identified. First, we should declare that we have previously presented this study at the Fetal Medicine Foundation congress 2024,^28^ through a poster, in which the C/S rate was not as specific as this study, due to clearing the data and adding the data after the presentation. Also, numerous instances of missing information in the documentation indicate a lack of attention to documentation by healthcare providers. Additionally, the absence of standardized diagnostic criteria for placental abnormalities (e.g. ultrasound and MRI protocols) across hospitals raises concerns regarding consistency. The variability in imaging practices may result in either underdiagnosis or overdiagnosis of conditions such as placenta accrete. Furthermore, it is essential to establish a registration system for these conditions to ensure persistent follow-up on documentation and data validity. In addition, focusing on Tehran limits generalizability to rural or other urban regions in Iran, where healthcare infrastructure, socioeconomic factors, and obstetric practices may differ significantly. For instance, Tehran’s tertiary care centers may handle more complex referrals, potentially inflating the reported incidence of PH compared to less-resourced areas.

## Conclusion

 Although this research provided a general overview of the influencing factors of PH through an investigation of cases in all private and public hospitals in Tehran, to make or modify health policies in this domain, a national-level study is warranted. We recommend considering regional variations in emergency management approaches, cultural, socioeconomic, and demographic characteristics, as well as the accessibility of essential resources, including blood banks and intensive care units.
